# Supplementary material for: The causal role of circulating inflammatory markers in osteoporosis: a bidirectional Mendelian randomized study
Source: Front Immunol. 2024 Jul 18;15:1412298. doi: 10.3389/fimmu.2024.1412298 (PMC11291241; doi:10.3389/fimmu.2024.1412298)
Supplement: Supplementary file 1 [file DataSheet_1.zip › supplementary material/CIM-OSTEOPOROSIS /GCST90274819.csv_leave_one_out.pdf]

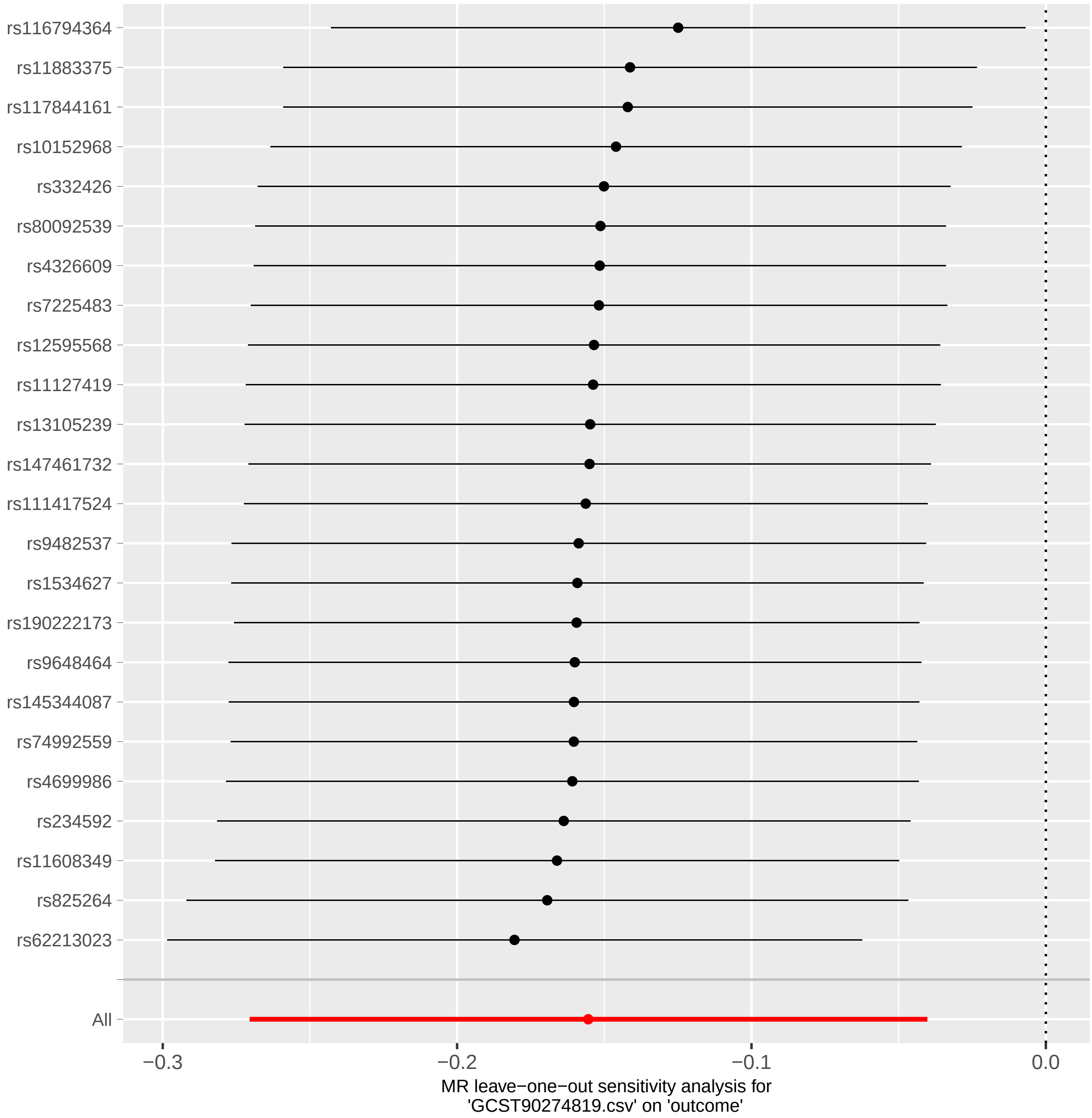
rs116794364
rs11883375
rs117844161
rs10152968
rs332426
rs80092539
rs4326609
rs7225483
rs12595568
rs11127419
rs13105239
rs147461732
rs111417524
rs9482537
rs1534627
rs190222173
rs9648464
rs145344087
rs74992559
rs4699986
rs234592
rs11608349
rs825264
rs62213023
All
−0.3
−0.2
−0.1
0.0
MR leave−one−out sensitivity analysis for
'GCST90274819.csv' on 'outcome'
